# Resveratrol Differentially Regulates NAMPT and SIRT1 in Hepatocarcinoma Cells and Primary Human Hepatocytes

**DOI:** 10.1371/journal.pone.0091045

**Published:** 2014-03-06

**Authors:** Susanne Schuster, Melanie Penke, Theresa Gorski, Stefanie Petzold-Quinque, Georg Damm, Rolf Gebhardt, Wieland Kiess, Antje Garten

**Affiliations:** 1 Center for Pediatric Research Leipzig, University Hospital for Children and Adolescents, Faculty of Medicine, University of Leipzig, Leipzig, Germany; 2 Department of General-, Visceral- and Transplantation Surgery, Charité University Medicine Berlin, Berlin, Germany; 3 Institute of Biochemistry, Faculty of Medicine, University of Leipzig, Leipzig, Germany; University of Navarra School of Medicine and Center for Applied Medical Research (CIMA), Spain

## Abstract

Resveratrol is reported to possess chemotherapeutic properties in several cancers. In this study, we wanted to investigate the molecular mechanisms of resveratrol-induced cell cycle arrest and apoptosis as well as the impact of resveratrol on NAMPT and SIRT1 protein function and asked whether there are differences in hepatocarcinoma cells (HepG2, Hep3B cells) and non-cancerous primary human hepatocytes. We found a lower basal NAMPT mRNA and protein expression in hepatocarcinoma cells compared to primary hepatocytes. In contrast, SIRT1 was significantly higher expressed in hepatocarcinoma cells than in primary hepatocytes. Resveratrol induced cell cycle arrest in the S- and G2/M- phase and apoptosis was mediated by activation of p53 and caspase-3 in HepG2 cells. In contrast to primary hepatocytes, resveratrol treated HepG2 cells showed a reduction of NAMPT enzymatic activity and increased p53 acetylation (K382). Resveratrol induced NAMPT release from HepG2 cells which was associated with increased NAMPT mRNA expression. This effect was absent in primary hepatocytes where resveratrol was shown to function as NAMPT and SIRT1 activator. SIRT1 inhibition by EX527 resembled resveratrol effects on HepG2 cells. Furthermore, a SIRT1 overexpression significantly decreased both p53 hyperacetylation and resveratrol-induced NAMPT release as well as S-phase arrest in HepG2 cells. We could show that NAMPT and SIRT1 are differentially regulated by resveratrol in hepatocarcinoma cells and primary hepatocytes and that resveratrol did not act as a SIRT1 activator in hepatocarcinoma cells.

## Introduction

Resveratrol, a dietary polyphenol, is reported to possess both chemopreventive and chemotherapeutic properties in several cancers [1]. In 1997, Jang and colleagues published a seminal paper reporting that resveratrol is able to inhibit carcinogenesis in all three stages (initiation, promotion and progression) [2]. Resveratrol was shown to inhibit cell proliferation, induce apoptosis and cell cycle arrest in different cancer types and cancer cell lines [3]–[9]. However, only one study compared the apoptotic effects of resveratrol on cancer and normal cells. Baarine *et al.* found apoptotic effects in murine tumoral cardiac cells which were absent in normal cardiomyocytes [8]. The molecular mechanisms are currently not completely understood. SIRT1 has originally been described as a target of resveratrol [10] although some of the data are still controversial, especially concerning resveratrol acting as SIRT1 activator in cancer cells [11]–[13]. SIRT1 belongs to the NAD (Nicotinamide adenine dinucleotide) dependent histone deacetylases, called sirtuins. SIRT1 is involved in many cellular pathways, such as cellular survival, apoptosis, cellular stress response and energy metabolism. An increased expression of SIRT1 has been reported in a variety of human cancers, including prostate, ovarian, gastric and colorectal cancer. The role of SIRT1 in tumorigenesis is still controversially discussed. SIRT1 has been shown to act as both tumor promoter and tumor suppressor [14], [15]. SIRT1 was shown to deacetylate the tumor-suppressor protein p53 on lysine residue 382 leading to its inhibition and subsequent tumorigenesis [16], [17]. Thus, the inhibition of SIRT1 would induce cell death of cancer cells by activating and acetylating p53.

It is known that cancer cells have increased energy demands because of their rapid cell proliferation and increased DNA repair [18]. NAD is required for both processes [19] and regulates crucial biological processes, including transcription, cell cycle progression, DNA repair and metabolic pathways [20], [21]. Therefore, cancer cells have a higher rate of NAD turnover than normal cells. The regeneration of intracellular NAD pools is regulated by NAMPT (Nicotinamide phosphoribosyltransferase). NAMPT can be found intracellularly (iNAMPT) and extracellularly (eNAMPT). However, neither structural differences between these forms nor the mechanism of NAMPT secretion are known so far. As an intracellular protein, NAMPT catalyses the rate-limiting step in the NAD salvage pathway starting from nicotinamide and yielding nicotinamide mononucleotide (NMN) which is then converted to NAD [22]–[25]. Some cancer cells maintain intracellular NAD levels by overexpressing NAMPT which has been shown in different cancer types, such as colorectal and breast cancer [26]–[28]. The expression and regulation of intracellular NAMPT in hepatocarcinoma cells has not been characterized so far. NAMPT inhibition by its highly specific inhibitor FK866 induces apoptosis and/or autophagy in tumor cells [29]–[32]. Moreover, previous studies pointed out that inhibition of NAMPT enzymatic activity by FK866 or inhibition of SIRT1 activity decreased proliferation and triggered cell death in cancer cells which was associated with increased acetylation of p53 (K382) [16], [17], [33], [34].

Here we investigated the molecular mechanisms of resveratrol-induced apoptotic effects on hepatocarcinoma cells and non-cancerous human hepatocytes and asked whether NAMPT and SIRT1 are differentially regulated in hepatocarcinoma cells and non-cancerous human hepatocytes.

## Materials and Methods

### Ethics Statement

Non-cancerous primary human hepatocytes were supplied by the “virtual liver” program (German Federal Ministry of Education and Research) and the non profit foundation HTCR, including the informed patient’s consent. The use of human hepatocytes for research purposes was approved by the local ethics committee of the Charité University Berlin. Written informed consent was obtained from all patients. The Charité University Berlin institutional review board specifically approved this study.

### Material

Cell culture media, supplements and antibiotics were obtained from PAA (Cölbe, Germany) or Invitrogen (Karlsruhe, Germany). Resveratrol (*trans* isomer), nicotinamide and camptothecin were purchased from Sigma-Aldrich (Munich, Germany). FK866 was kindly provided by TopoTarget A/S, Copenhagen, Denmark. EX527 was obtained from Cayman Chemical (Ann Arbor, USA), InSolution Trichostatin A (TSA) and etoposide were purchased from Merck Millipore (Darmstadt, Germany). Flag-SIRT1 expression vector was obtained from Addgene (Addgene plasmid 1791) [35].

### Cell Culture

HepG2 cells were purchased from Leibniz Institute DSMZ (German Collection of Microorganisms and Cell cultures) and Hep3B cell were kindly provided by Prof. Dr. Kurt Engeland (Molecular Oncology, Medical School, University of Leipzig). Cells were maintained in MEM medium supplemented with 10% fetal bovine serum (FBS) and 2 mmol/L glutamine and 100 IU penicillin and 100 µg/mL streptomycin. Primary human hepatocytes were isolated and cultured essentially as described [36]. Cells were seeded in Williams’ Medium E containing 2 mmol/L glutamine, 10^−7^ mol/L dexamethansone, 100 IU penicillin and 100 µg/mL streptomycin and 10% FBS. All cells were grown at 37°C in a humidified atmosphere of 95% air and 5% CO_2_.

### Cell Treatments

Resveratrol was dissolved in 100% ethanol to create a stock solution of 100 mM. Cells were stimulated with 10/25/50/100 µM resveratrol and the equivalent amount of solvent control (ethanol) to exclude solvent-mediated effects. To inhibit SIRT1 and deacetylases other than histone deacetylases class III, we used the compound EX527 [20 µM], a cell-permeable selective inhibitor of SIRT1 dissolved in DMSO [37] and 1 µM of TSA which were added to the incubation medium.

### Measurement of Cell Viability and Apoptosis

To investigate the effects on proliferation and cell viability, we used the commercial Cell Proliferation Reagent WST-1 (Roche, Grenzach-Wyhlen, Germany) and measured absorbance at 450 nm. To evaluate the effects of resveratrol on apoptosis the number of apoptotic cells was measured by flow cytometry using the FITC Annexin V Apoptosis Detection Kit (BD Pharmingen, Franklin Lakes, USA). Adherent and floating cells were used. 5–10 µL of Annexin V-FITC (An) and 2 µL of propidium iodide (PI) were added to the cell suspension. Samples were analysed using a Beckton-Dickinson FACS LSRII. As positive control, apoptosis was induced via camptothecin [2 µM] and etoposide [85 µM] for 24 h. An+ and double-stained An+/PI+ cells were considered apoptotic. To exclude cytotoxic effects of resveratrol, we used supernatant of HepG2 cells and primary human hepatocytes to measure the release of the enzyme, adenylate kinase, from damaged cells. Therefore, we used the ToxiLight™ Non-destructive Cytotoxicity BioAssay Kit (Lonza, Cologne, Germany).

### Cell Cycle Distribution Analysis

PI staining was used to analyse DNA content and cell cycle distribution. After cell treatment, adherent and floating cells were harvested and fixed with 2 mL of 70% ethanol (4°C). The cell pellet was resuspended in 50 µL PBS with 3.3 µL RNase A [30 mg/mL], 450 µL FACS-buffer (PBS+2% FBS) and PI [50 µg/mL] were added to the flow cytometry tubes. Cells were analysed using a Beckton-Dickinson FACS LSRII by measuring the PI signal in the FL2 channel.

### Reverse Transcription-quantitative Real-time PCR (RTqPCR)

To measure mRNA expression, total RNA was extracted using the RNeasy Mini Kit (Qiagen, Hilden, Germany) according to the manufacturer’s instructions. Reverse transcription was performed using 200 U M-MLV reverse transcriptase (Invitrogen, Karlsruhe, Germany) per 500 ng or 1 µg total RNA with random hexamer [p(dN)6] primers. mRNA expression was quantified by real-time PCR with TaqMan probe based (Eurogentec, Cologne, Germany) or SYBR green based (Primerdesign, Southampton, UK) gene expression assay on the ABI 7500 Sequence Detection System (Applied Biosystems, Darmstadt, Germany). The housekeeping genes *TATA-box-binding protein* (TBP), *hypoxanthine phosphoribosyltransferase* (HPRT) or beta-*ACTIN* were quantified simultaneously. Sequence information of primers and probes are given in Table 1. For standardization of gene expression, the target gene amount was normalized to the mean of the housekeeping gene expression in each sample.

**Table 1:** Sequences of Primer and Probes used for *real-time* PCR (TaqMan).

Target	Forward Primer	Reverse Primer	Probe
*NAMPT*	GCA GAA GCC GAG TTC AAC ATC	TGC TTG TGT TGG GTG GAT ATT G	TGG CCA CCG ACT CCT ACA AGG TTA CTC AC
*beta-ACTIN*	CGA GCG CGG CTA CAG CTT	CCT TAA TGT CAC GCA CGA TTT	ACC ACC ACG GCC GAG CGG
*TBP*	TTG TAA ACT TGA CCT AAA GAC CAT TGC	TTC GTG GCT CTC TTA TCC TCA TG	AAC GCC GAA TAT AAT CCC AAG CGG TTT G
*HPRT*	GGC AGT ATA ATC CAA AGA TGG TCA A	GTC TGG CTT ATA TCC AAC ACT TCG T	CAA GCT TGC TGG TGA AAA GGA CCC C
*p21*	CGAAGTCAGTTCCTTGTGGAG	CATGGGTTCTGACGGACAT	–

*NAMPT (*nicotinamide phosphoribosyltransferase, also known as PBEF, visfatin); *p21*; housekeeping genes *beta-ACTIN*, *TBP* (TATA-box-binding protein) and *HPRT* (hypoxanthine phophoribosyltransferase).

### Protein Extraction and Immunoblotting

For protein analyses, cells were lysed in modified RIPA buffer (50 mM TrisHCl pH 7.4; 1% NP-40; 0.25% sodium deoxycholate; 1×Roche complete proteases inhibitor cocktail; 1 mM EDTA; 1 mM sodium orthovanadate; 1 mM sodium fluoride; 5 mM nicotinamide; 5 µM TSA, 1 mM sodium butyrate) and separated by SDS-PAGE (8–15%). Protein concentration of lysates was measured by BCA protein assay (Pierce, Thermo Scientific). After transfer to nitrocellulose membranes (Millipore, Bedford, MA, USA), blots were blocked with 5% (w/v) non-fat dry milk in 1×TBS buffer containing 0.1% Tween 20 (TBS-T). Primary antibodies used for immunoblotting included anti-NAMPT clone OMNI 379 (1∶5000) (Cayman Chemical, Ann Arbor, MI, USA), anti- acetylated p53 (K382) (1∶1000), anti-p53 (1∶1000), anti-p53 (1C12) (1∶1000), anti-phospho-p53 (Ser15) (1∶1000), anti-SIRT1 (D379) (1∶1000), anti-p21 (1∶1000), anti-Bax (1∶1000), anti-caspase3 (1∶500), anti- cleaved caspase3 (1∶500) (Cell Signaling, Beverly, MA, USA) and anti-GAPDH (MerckMillipore, Schwalbach, Germany). Secondary antibodies were purchased from DAKO (Hamburg, Germany). Immunoblotting for GAPDH was performed to verify equivalent amounts of loaded protein. Detection was performed using enhanced chemiluminescence. Densitometric analysis was performed using ImageJ 1.41 Software (NIH, USA).

### Measurement of NAMPT Release

NAMPT concentration in supernatants of HepG2 cells and primary hepatocytes was quantified using the human extracellular NAMPT/PBEF/Visfatin ELISA Kit (AdipoGen Inc., Seoul, South Korea), respectively, according to manufacturer’s instructions. NAMPT concentration was normalised to the corresponding total protein amount in each sample. For semiquantitative measurements, NAMPT levels were detected by using supernatant of cultured cells for Western Blot analysis.

### NAMPT Enzymatic Activity

NAMPT activity was measured by the conversion of ^14^C- labelled nicotinamide to ^14^C-NMN using a method previously described [38]. For preparation of lysates, cells were harvested and resuspended in 100 µL of 0.01 mol/L sodium phosphate buffer, pH 7.4, frozen at –80°C for 24 h and thawed at room temperature. Cell debris was removed by centrifugation at 23,000 rcf, 90 min at 0°C. Protamine sulphate solution (1% in NaHPO4 buffer) was added to the supernatant (70 µL/mL supernatant) to precipitate DNA by incubation on ice for 15 min. After centrifugation at 23,000 rcf, 30 min at 0°C, aliquots of the supernatant were stored at –80°C. Lysates (50 µg) were added to 50 µL reaction mix (50 mmol/L TrisHCl; 2 mmol/L ATP; 5 mmol/L MgCl2; 0.5 mmol/L PRPP; 6.2 µmol/L ^14^C-nicotinamide; American Radiolabelled Chemicals, St. Louis; MO, USA) and incubated at 37°C for 1 h. Optimal conditions for the NAMPT activity assay (amount of total protein, incubation time, pH value) were determined (Fig. S1A,B,C). For measuring extracellular NAMPT activity we used supernatant of HepG2 cells and concentrated it 80-fold using Amicon Ultra Centrifugal Filter Units (Ultracel-50k) (Millipore). Then, 10 µl of concentrated supernatant was used for the enzyme assay reaction mix and incubated for 2 h at 37°C. The NAMPT enzymatic reaction was terminated by mixing with 2 mL of acetone. The mixture was then transferred onto acetone-pre-soaked glass microfiber filters (GF/A Ø 24 mm; Whatman, Maidstone, UK). After rinsing with 2×1 mL acetone, filters were dried, transferred into vials with 6 mL scintillation cocktail (Betaplate Scint, PerkinElmer, Waltham, MA, USA) and radioactivity of ^14^C-NMN was quantified in a liquid scintillation counter in counts per minute (cpm) (Wallac 1409 DSA, PerkinElmer). NAMPT activity was normalised to total protein concentration as measured by the BCA protein assay. The validity of the assay was evaluated by adding the specific NAMPT inhibitor, FK866 (Fig. S1C). FK866 induced a dose-dependent decrease in NAMPT activity with an IC_50_ value of 8.2 nM.

### NAD Measurements

Concentrations of NAD from whole-cell extracts were quantified by High-performance liquid chromatography (HPLC) and the NAD/NADH assay kit (EnzyChrom NAD/NADH Assay Kit, Biotrend, Köln, Germany), applied according to manufacturer’s instructions. HPLC analysis was performed with Chromaster Purospher STAR RP-18 endcapped 3 µm Hibar RT 150-3 HPLC column (Merck). Briefly, cultured cells were extracted in 1 M HClO_4_ and neutralized in 3 M K_2_CO_3_ on ice as described previously [39]. After centrifugation for 10 min at 18,000 rcf (4°C), the supernatant was filtered and loaded onto the column. For NAD measurement, the HPLC was run at a flow rate of 0,4 ml/min with 100% buffer A from 0–5 min, a linear gradient to 95% Buffer A/5% Buffer B (100% methanol) from 5–6 min, 95% Buffer A/5% Buffer B from 6–11 min, a linear gradient to 85% Buffer A/15% Buffer B from 11–12 min, 85% Buffer A/15% Buffer B from 12–16 min, and a linear gradient to 100% Buffer A from 16–17 min. NAD was eluted as a sharp peak at 15 min and quantitated based on the peak area compared to a standard curve and normalised to protein content of cultured cells.

### Plasmid Transfection

Transfection was conducted using NEON Transfection System (100 µl Kit, invitrogen) according to the manufacturer’s manual. Briefly, HepG2 cells were splitted 1∶3 one day before transfection. Cells were transiently transfected with pECE-Flag-SIRT1 (2 µg DNA/0.5×10^6^ cells) or the empty vector (mock-control). After 24 h of transfection, medium was changed for a further 24 h resveratrol-containing medium at 37°C.

### Statistical Analyses

Data are presented as mean ± SEM. Data were analysed for statistical significance by one-way analysis of variance (ANOVA) followed by Bonferroni post hoc test. Differences between two groups were evaluated using unpaired Student’s *t*-test. All analyses were performed using GraphPad Prism 5 software (GraphPad Software, Inc., San Diego, USA). The level of significance for all comparisons was set at p<0.05.

## Results

### NAMPT and SIRT1 are Differentially Expressed in Hepatocarcinoma Cells and Primary Human Hepatocytes

**Figure 1:**
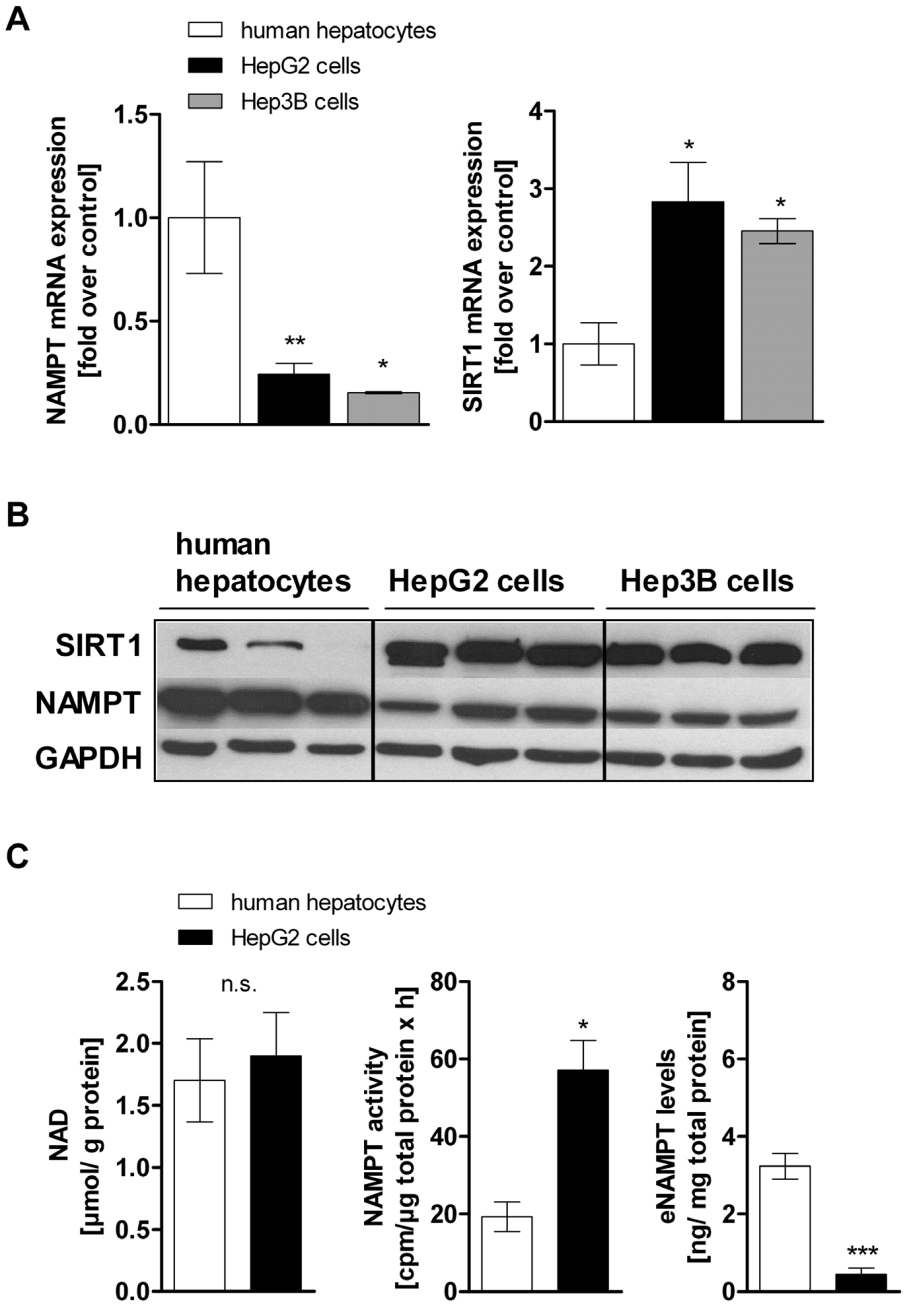
NAMPT and SIRT1 expression in hepatocarcinoma cells and primary human hepatocytes. A) mRNA expression and B) protein expression of NAMPT and SIRT1 in primary human hepatocytes (n = 7), HepG2 cells (n = 8) and Hep3B cells (n = 3). Representative Western Blot is shown out of three independent experiments. Measurement of C) intracellular NAD levels (left panel, primary hepatocytes n = 4, HepG2 cells n = 6), basal NAMPT enzymatic activity (middle panel, primary hepatocytes n = 3, HepG2 cells n = 4) and extracellular NAMPT (eNAMPT) levels (right panel, primary hepatocytes n = 3, HepG2 cells n = 6) in primary human hepatocytes and HepG2 cells. Data are shown as mean± SEM. Difference between two groups was evaluated using unpaired Student’s *t*-test (*p<0.05, **p<0.01, ***p<0.001).

The expression levels of NAMPT and SIRT1 were evaluated using qPCR and Western Blot analysis. Our data revealed that NAMPT expression is lower in HepG2 (−75.6%±5.2%) and Hep3B cells (−84.6%±0.5%) compared to non-cancerous primary human hepatocytes (Fig. 1A,B). In contrast, the NAD-dependent deacetylase SIRT1 is significantly higher expressed in both cancer cell lines compared to primary human hepatocytes (HepG2 cells 2.8-fold, Hep3B cells 2.5-fold) (Fig. 1A,B). Intracellular NAD levels in HepG2 cells and primary hepatocytes were not significantly different (HepG2 cells 1.9±0.3 µmol NAD/g protein compared to 1.7±0.3 µmol NAD/g protein in primary human hepatocytes) (Fig. 1C, left panel). A comparison of the NAMPT enzymatic activity in HepG2 cells and primary human hepatocytes showed a 3-fold higher (p<0.05) enzymatic activity of NAMPT in HepG2 cells (57.2±7.7 cpm/µg protein×h) than in primary human hepatocytes (19.3±3.8 cpm/µg protein×h) (Fig. 1C, middle panel). Additionally, we measured higher eNAMPT levels in the supernatant of primary human hepatocytes (3.2±0.3 ng/mg protein) than in HepG2 cells (0.4±0.2 ng/mg protein) (Fig. 1C, right panel).

### Resveratrol Induces Cell Cycle Arrest and Apoptosis in Hepatocarcinoma Cells

**Figure 2:**
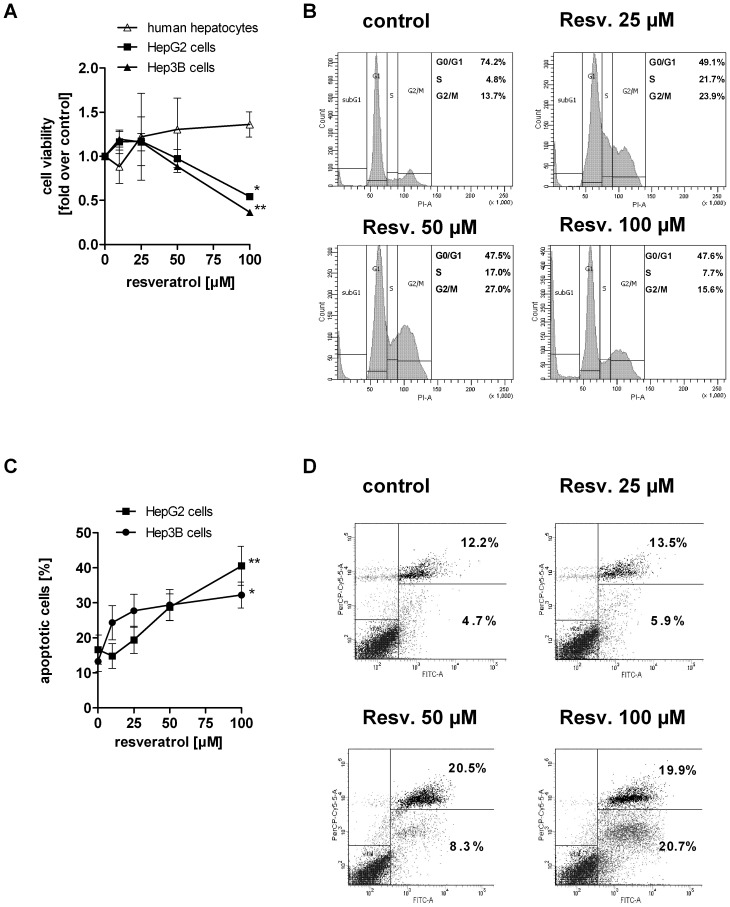
Resveratrol reduces cell proliferation and induces cell cycle arrest and apoptosis in hepatocarcinoma cells which is absent in primary human hepatocytes. Cell viability of A) primary human hepatocytes (n = 2), HepG2 and Hep3B cells (n = 3) after stimulation with resveratrol for 24 h. Data were normalised to serum-free medium control which was set 1. B) Cell cycle distribution of HepG2 cells treated with resveratrol for 24 h. A representative result is shown out of three independent experiments. A representative dot plot is given in Fig. S2B. C) Annexin V/PI apoptosis assay of HepG2 (n = 3) and Hep3B cells (n = 3) treated with resveratrol for 24 h. D) A representative dot plot of the Annexin/PI staining in HepG2 cells is shown including the mean percentage of An+ and double An+/PI+ cells of three independent experiments. Data are shown as mean± SEM and statistical analysis was performed using one-way ANOVA and the Bonferroni post hoc test (*p<0.05; **p<0.01 compared to serum-free medium).

Resveratrol has been shown to induce growth arrest and apoptosis in many different cancer cell lines. In the present study, we wanted to investigate whether the effects of resveratrol are p53-dependent. Therefore, we used HepG2 cells, known to be p53 wild-type, Hep3B cells - a p53 deficient cell line due to a deletion of the p53 gene, and primary human hepatocytes as non-cancerous hepatocyte control. Cells were treated with resveratrol as described above. After 24 h, hepatocarcinoma cells showed a dose-dependent decrease in viability (Fig. 2A). Resveratrol [100 µM] markedly decreased cell viability by 45.8±2.7% (p<0.05) in HepG2 cells and by 63.7±3.4% (p<0.01) in Hep3B cells (Fig. 2A). Primary human hepatocytes treated with the same concentrations of resveratrol exhibited no significant changes in viability (Fig. 2A). To investigate the cause of cell viability reduction by resveratrol, we analysed cell cycle distribution. As shown in Fig. 2B, resveratrol [25, 50 µM] caused an increase of cells in the S-phase ([con] 4.7±0.6%, [25 µM] 21.7±5.1%, [50 µM] 17.0±2.6%, p<0.05) and in the G2/M-phase ([con] 13.7±1.8%, [25 µM] 23.9±4.2%, [50 µM] 27.0±6.1%, p<0.05) and a corresponding decrease of cells in the G1-phase. The cell cycle distribution was not significantly modified in p53-deficient Hep3B cells (Fig. S2A), which indicates that the resveratrol-induced cell cycle arrest is mediated by a functional p53. However, in both hepatocarcinoma cell lines apoptotic mechanisms were activated. As indicated in Fig. 2C, stimulation with increasing concentrations of resveratrol led to a dose-dependent increase in the number of apoptotic cells in HepG2 (Fig. 2C,D) and Hep3B cells (Fig. 2C). At 100 µM resveratrol, the percentage of apoptotic cells in HepG2 cells and Hep3B cells increased to 40.6±5.6% (p<0.01) and to 32.2±3.7% (p<0.05), respectively.

### p53 and Caspase-3 are Involved in Resveratrol-mediated Apoptotic Effects

**Figure 3:**
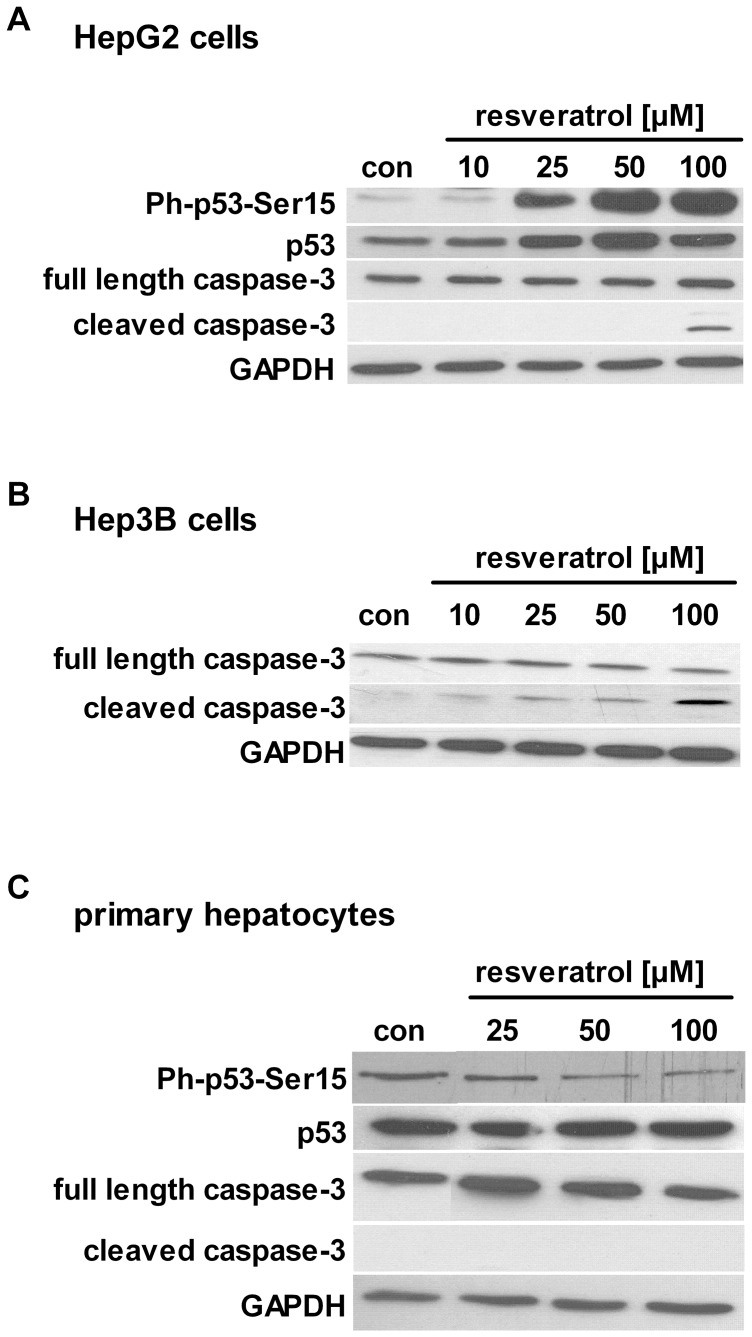
Resveratrol activates apoptotic mechanisms in hepatocarcinoma cells. Cells were treated with resveratrol or serum-free medium (con) for 24 h. Activation of p53 through phosphorylation at serine residue 15 and cleavage of caspase-3 in A) HepG2 cells, B) Hep3B cells and C) primary human hepatocytes were analysed by Western Blot. GAPDH was used as loading control. One representative blot out of at least 3 independent experiments is shown.

In HepG2 cells, resveratrol increased the phosphorylation of p53 at residue serine 15 in a dose-dependent manner (Fig. 3A). At high concentration of resveratrol [100 µM], we found increased cleavage of caspase-3 (Fig. 3A). The activation of caspase-3 by resveratrol was also increased in p53-deficient Hep3B cells even at lower concentration [25, 50 µM] (Fig. 3B). These results indicate that resveratrol induces caspase-3 activation in a p53-independent manner. Then, we stimulated primary human hepatocytes with the same concentrations of resveratrol and found no induction of apoptosis (Fig. 3C) or cytotoxicity (Fig. S3A).

### Inhibition of NAMPT and SIRT1 Activity in Hepatocarcinoma Cells Induces Growth Arrest and Apoptosis

**Figure 4:**
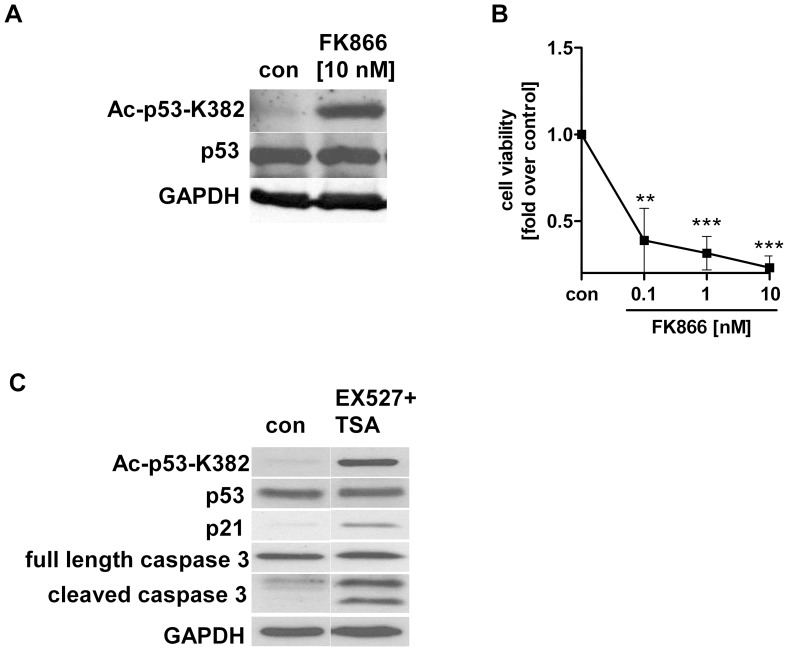
Effects of FK866 and EX527 on p53 acetylation and cell viability in HepG2 cells. Cells were stimulated with FK866 [10 nM] or EX527+TSA [20 µM EX527+1 µM TSA] in serum-free medium (con). Cells treated with A) FK866 and expression of acetylated p53 (K382) after 24 h. B) Cell viability of HepG2 cells after stimulation with FK866 for 48 h measured by WST-1 assay (n = 4). Data were normalised to serum-free medium (con) which was set 1 (**p<0.01; ***p<0.001 compared to serum free medium). C) Expression of acetylated p53 (K382), p21 protein and cleavage of caspase-3 were analysed in HepG2 cells treated with EX527+TSA for 24 h. GAPDH was used as loading control. One representative blot out of 3 independent experiments is shown.

Several studies have shown that the NAD metabolism is essential for cancer cell survival and proliferation [40]–[42]. However, little is known about the effects of resveratrol on NAMPT and SIRT1 activity in hepatocarcinoma cells. Human SIRT1 targets and deacetylates the p53 tumor suppressor protein [16], [17], [34]. Therefore, we investigated whether a specific inhibition of NAMPT and SIRT1 would affect cell survival and apoptotic mechanisms. We used the specific NAMPT inhibitor FK866 and the SIRT1 inhibitor EX527 [34]. FK866 increased p53 acetylation (K382) (Fig. 4A) and reduced HepG2 cell viability after 48 h (Fig. 4B). Cells treated with the SIRT1 inhibitor, EX527, showed also increased p53 acetylation (K382), enhanced expression of p53 downstream target p21/WAF1/Cip1 and activation of caspase-3 (Fig. 4C).

### Resveratrol Differentially Regulates NAMPT Enzymatic Activity in Hepatocarcinoma Cells and Primary Human Hepatocytes

**Figure 5:**
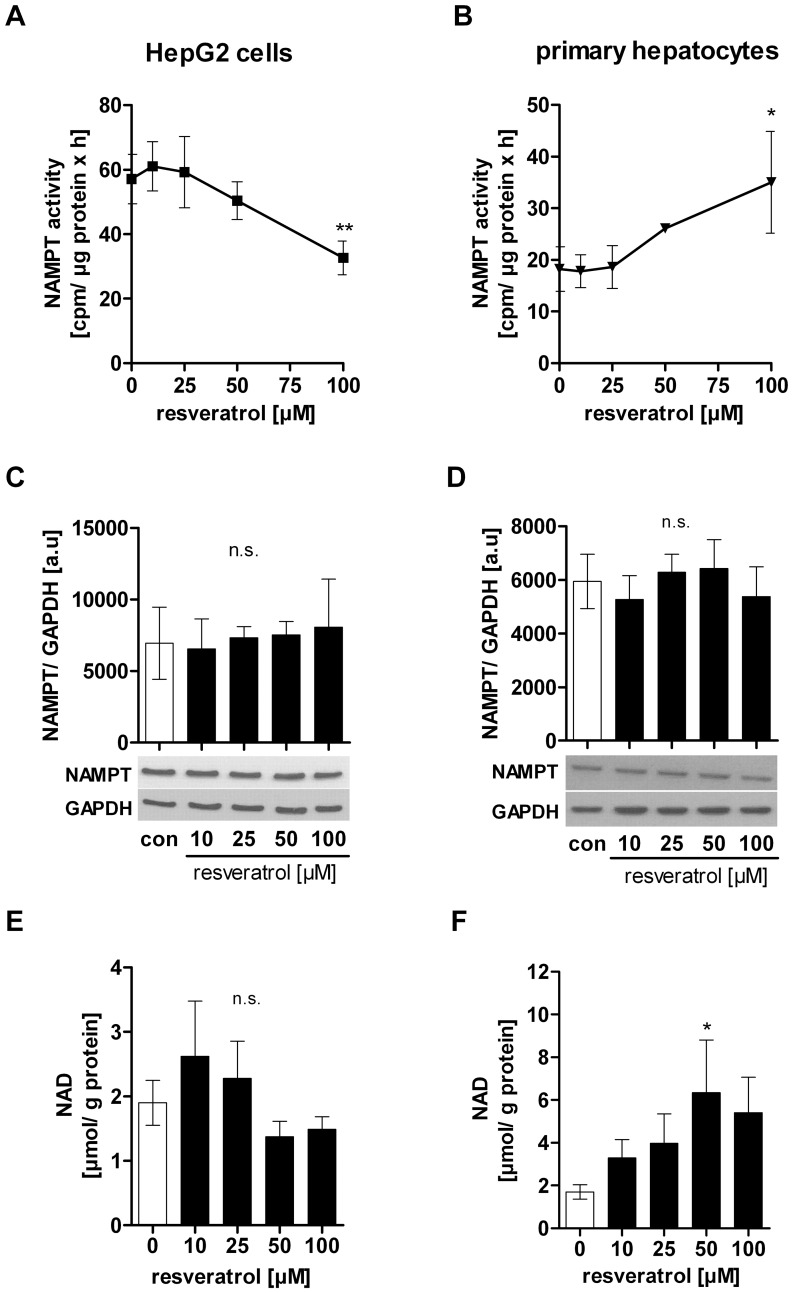
Resveratrol differentially regulates NAMPT and NAD levels in HepG2 cells and primary human hepatocytes. Cells were stimulated with resveratrol or serum-free medium (con) for 24 h. For measuring NAMPT enzymatic activity in A) HepG2 cells and (n = 4) B) primary human hepatocytes (n = 3), 50 µg of protein lysate was used for the assay and incubated for 1 h. Counts (cpm) were normalised to µg total protein. Lysates from C) HepG2 cells (n = 3) and D) primary human hepatocytes (n = 3) were used to measure NAMPT protein levels by Western Blot. Determination of intracellular NAD levels in E) HepG2 cells (n = 6) and F) primary human hepatocytes (n = 4). NAD levels were normalised to total protein amount in each sample.

Based on our findings that an inhibition of NAMPT and SIRT1 activity induced growth arrest and apoptosis in hepatocarcinoma cells we then asked whether resveratrol would also affect NAMPT enzyme activity as well as intracellular NAD levels. We found that resveratrol differentially regulated NAMPT activity in hepatocarcinoma cells (Fig. 5A) and primary hepatocytes (Fig. 5B) without affecting NAMPT protein expression (Fig. 5C,D). We measured a dose-dependently decreased NAMPT activity in HepG2 cells ([100 µM] −38.9±14.0%, p<0.01) (Fig. 5A) and in Hep3B cells ([100 µM] −38.5±9.4%, p<0.05) (Fig. S4). In contrast, NAMPT enzymatic activity in primary hepatocytes significantly increased by +64.7±13.8% (p<0.05) after stimulation with 100 µM resveratrol (Fig. 5B). We then measured the NAD level after resveratrol treatment in HepG2 cells and found a trend towards reduction ([con] 2.0±0.4 µmol NAD/g total protein, [100 µM] 1.5±0.2 µmol NAD/g total protein) (Fig. 5E). In contrast, intracellular NAD levels in primary hepatocytes were increased by resveratrol ([con] 1.7±0.3 µmol NAD/g total protein, [50 µM] 6.4±2.5 µmol NAD/g total protein (p<0.05), [100 µM] 5.4±1.7 µmol NAD/g total protein) (Fig. 5F).

### Resveratrol Differentially Regulates p53 Acetylation and SIRT1 Protein in Hepatocarcinoma Cells and Primary Human Hepatocytes

**Figure 6:**
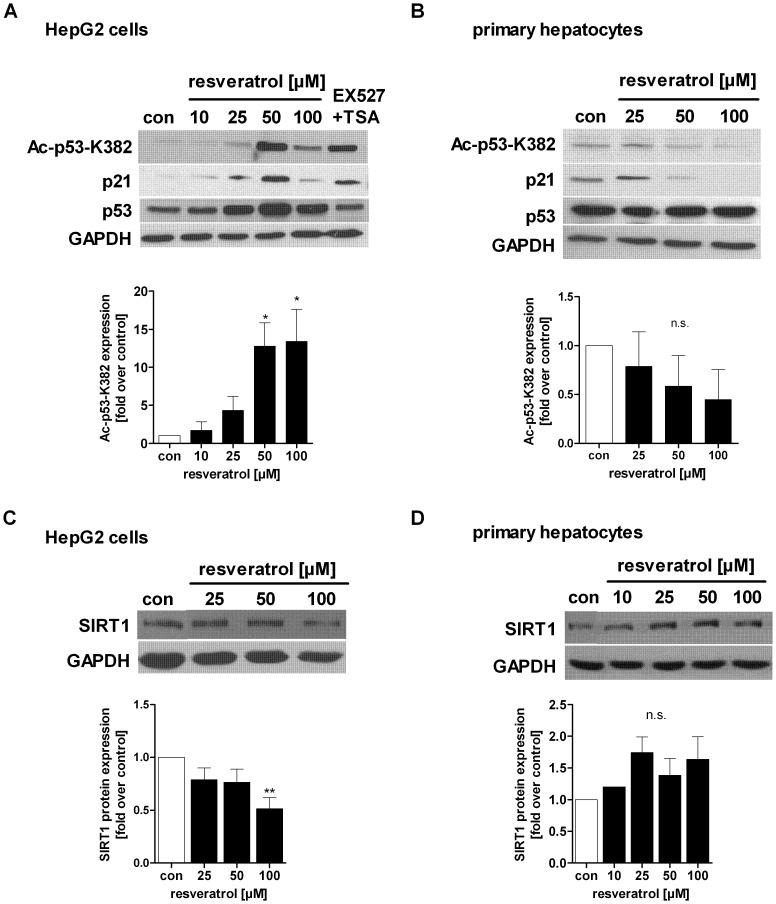
Resveratrol differentially regulates p53 acetylation and SIRT1 protein level in HepG2 cells and primary human hepatocytes. Acetylation of p53 (K382) in A) HepG2 cells (n = 4) and B) primary human hepatocytes (n = 3) was evaluated by Western Blot. Densitometric analysis of at least three independent experiments is shown. Data are represented as mean± SEM and statistical analysis was performed using one-way ANOVA and the Bonferroni post hoc test (*p<0.05, n.s. not significant). As a downstream target of acetylated and activated p53, the expression of p21 was analysed by Western Blot. As positive control for SIRT1 inhibition, EX527+TSA was used. SIRT1 protein expression was analysed by Western Blot in C) HepG2 cells and D) primary hepatocytes and densitometric analysis was performed. GAPDH was used as loading control, respectively. One representative blot out of at least 3 independent experiments is shown.

We further addressed whether resveratrol could influence p53 acetylation at lysine residue 382, a main target site of SIRT1 [16], [17], [34], and demonstrated that resveratrol treatment of HepG2 cells increased acetylation of p53 ([50 µM] 12.8-fold, [100 µM] 13.4-fold) (Fig. 6A). As positive control for SIRT1 inhibition, we used the specific SIRT1 inhibitor EX527 [34], [37] (Fig. 6A). In contrast, primary human hepatocytes from different donors showed a trend towards reduced p53 acetylation after resveratrol stimulation (Fig. 6B). Since the acetylation of p53 activates its transcriptional activity, we analysed the expression of the p53 downstream target p21/WAF1/Cip1, which functions as a regulator of cell cycle progression. In correspondence to the acetylation state of p53 we found increased expression of p21 mRNA (Fig. S5A) and protein in HepG2 cells (Fig. 6A). Primary human hepatocytes from different donors showed variable results with either no changes in p21 protein expression or a p21 down regulation (Fig. 6B, Fig. S5B). Nonetheless, we can exclude an activation of p53 in primary human hepatocytes. Furthermore, SIRT1 protein levels in HepG2 cells were reduced at 100 µM resveratrol (p<0.01) (Fig. 6C) whereas primary human hepatocytes showed a trend towards increased SIRT1 protein expression at the same dose of resveratrol (Fig. 6D). Due to the variability of primary hepatocytes the changes were not significant.

### Resveratrol Induces NAMPT Release in HepG2 Cells

**Figure 7:**
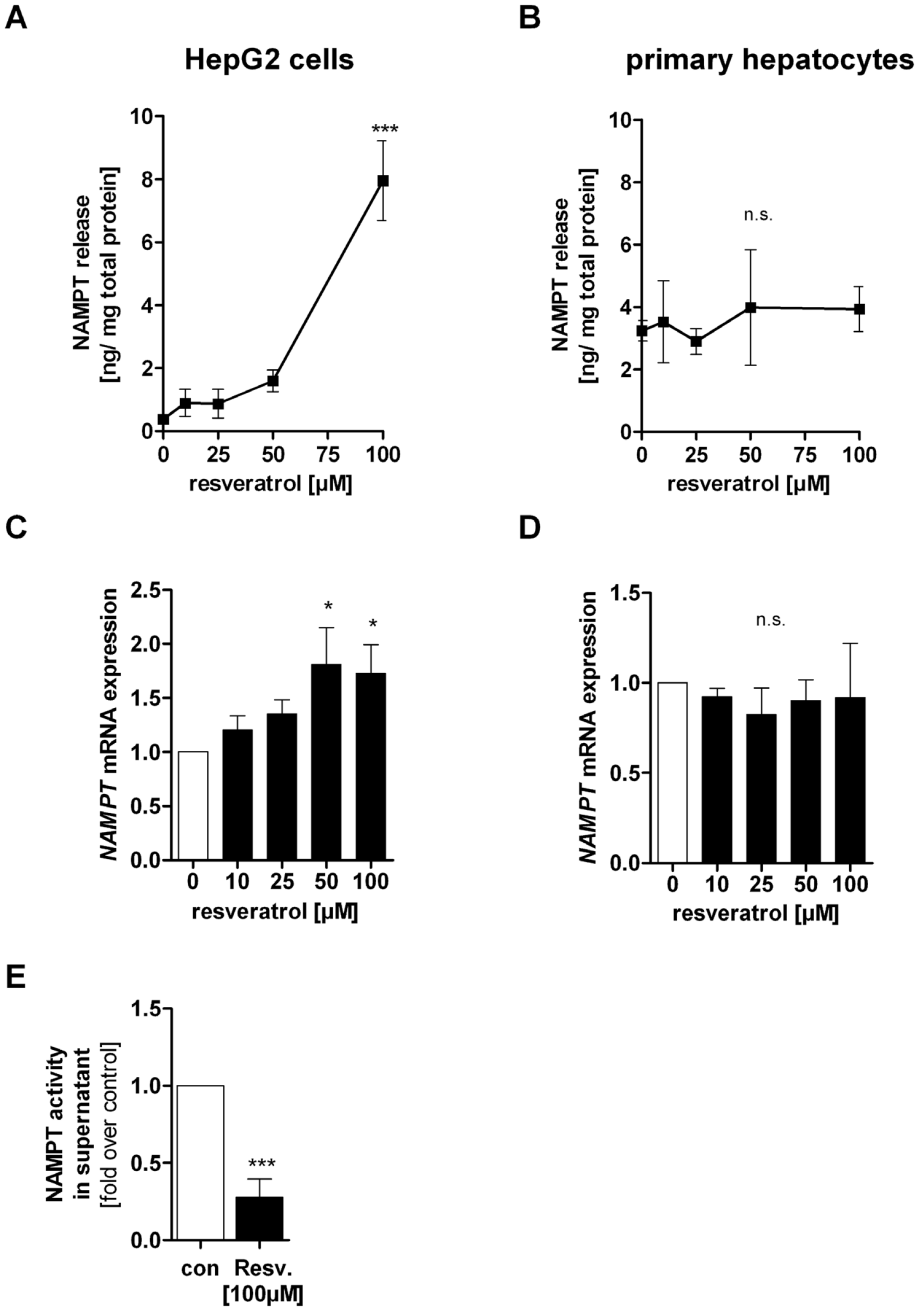
Effects of resveratrol on NAMPT release and NAMPT mRNA expression. Cells were stimulated with resveratrol in serum-free medium for 24 h. Supernatants of resveratrol treated A) HepG2 cells (n = 7) and B) primary human hepatocytes (n = 3) were used for quantifying extracellular NAMPT protein amount using a specific eNAMPT ELISA. eNAMPT protein concentration was normalised to the total protein amount. *NAMPT* mRNA expression in resveratrol treated C) HepG2 cells (n = 5) and D) primary human hepatocytes (n = 4) was quantified by qRT-PCR and normalised to housekeeping genes. *NAMPT* gene expression was then related to its expression in serum-free control medium (0), which was set 1. Data are represented as mean± SEM and statistical analysis was performed using one-way ANOVA and the Bonferroni post hoc test (*p<0.05; ***p<0.001; n.s. not significant). E) Supernatant of resveratrol [100 µM] or serum-free medium (con) treated HepG2 cells was used to measure NAMPT enzymatic activity and extracellular NAMPT protein levels. Counts (cpm) measured by NAMPT enzyme assay were referred to densitometric data of NAMPT protein levels in the supernatant of the same sample. Data were then normalised to serum-free control medium which was set 1. Data are shown as mean± SEM. The difference between these two groups was evaluated using unpaired Student’s *t*-test (***p<0.001).

Since NAMPT was found to be released from hepatocytes [43] we determined NAMPT concentrations in supernatants from resveratrol-treated HepG2 cells and primary hepatocytes. We measured significantly increased amounts of extracellular NAMPT in the supernatant of HepG2 cells treated with 50 µM (4-fold) and 100 µM (19.8-fold) resveratrol (Fig. 7A) ([con] 0.4±0.2 ng NAMPT/mg total protein, [50 µM] 1.6±0.7 ng NAMPT/mg total protein, [100 µM] 7.9±1.3 ng NAMPT/mg total protein, p<0.001). We postulated that *NAMPT* mRNA expression may be increased following resveratrol exposure in HepG2 cells to maintain a steady-state of intracellular NAMPT protein level. We found a significantly increased *NAMPT* gene expression after stimulation with 50 µM (1.8-fold, p<0.05) and 100 µM (1.7-fold, p<0.05) resveratrol in HepG2 cells (Fig. 7C). NAMPT release and *NAMPT* mRNA expression in primary human hepatocytes were not influenced by resveratrol (Fig. 7B,D). In parallel, a cytotoxicity assay was performed to verify that the increase of extracellular NAMPT levels was not due to leakage from damaged cells (Fig. S3B). We then asked at which time point NAMPT release from HepG2 cells started. We found that there was a time- and dose- dependent release of NAMPT already starting after 6 h of resveratrol exposure (Fig. S3C). NAMPT is known as a protein with dual function- an enzyme and a cytokine-like function. We asked whether NAMPT that is released after resveratrol exposure could lack NMN biosynthetic action. We found a remarkable decrease in extracellular NAMPT activity by 72.3±11.9% (p<0.001) compared to control cells in serum-free medium (Fig. 7E).

### NMN does not Protect Against Resveratrol- induced Apoptosis in Hepatocarcinoma Cells

Next, we investigated whether NMN would be able to ameliorate resveratrol-mediated effects in HepG2 cells. Interestingly, NMN did not protect from resveratrol-induced cell cycle arrest and apoptosis in hepatocarcinoma cells (Fig. S6A,B,C,D). Further, NMN was not able to abrogate p53 hyperacetylation after resveratrol treatment and to decrease resveratrol-induced NAMPT release in HepG2 cells (Fig. S6E,F).

### SIRT1 Inhibition Decreases NAMPT Activity and Induces NAMPT Release

**Figure 8:**
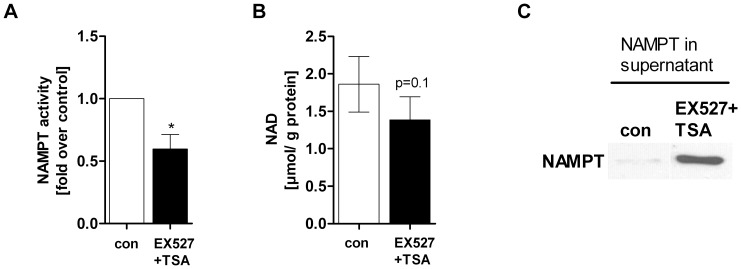
SIRT1 inhibition downregulates NAMPT activity and induces NAMPT release in HepG2 cells. HepG2 cells were treated with EX527+TSA [20 µM EX527+1 µM TSA] or serum-free medium (con) for 24 h. Measurement of A) NAMPT enzymatic activity (n = 3). Counts (cpm) were normalised to µg total protein in each sample (*p<0.05). B) NAD level were determined by HPLC (n = 5) and normalised to total protein amount in each sample. C) Supernatant of EX527 treated HepG2 cells was used for determination of eNAMPT level. One representative Western blot out of 3 independent experiments is shown.

Given that resveratrol increased p53 acetylation (K382), downregulated NAMPT activity and induced NAMPT secretion, we asked whether an inhibition of SIRT1 by EX527 would exert the same effects. Indeed, our data revealed that HepG2 cells treated with EX527 showed the same cellular responses as cells stimulated with resveratrol, such as decreased NAMPT enzymatic activity (−40.3±11.5%, p<0.05) (Fig. 8A) and slightly reduced intracellular NAD level (Fig. 8B). Further, as observed in resveratrol-treated HepG2 cells, extracellular NAMPT levels were increased upon SIRT1 inhibition (Fig. 8C).

### SIRT1 Overexpression Abrogated Resveratrol- induced p53 Hyperacetylation, NAMPT Release and S-phase Arrest

**Figure 9:**
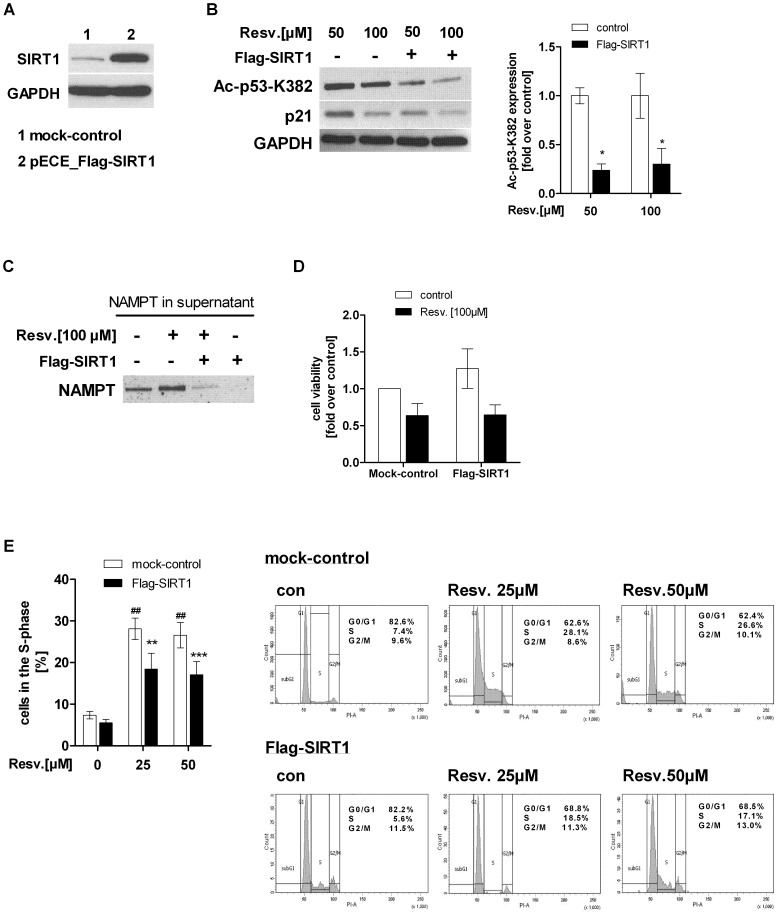
SIRT1 overexpression in HepG2 cells reversed resveratrol-induced SIRT1 inhibition, NAMPT release and S-phase arrest. A) SIRT1 was transiently overexpressed in HepG2 cells [2.0 µg plasmid/0.5x10^6^ cells] using the expression vector pECE_Flag-SIRT1 from addgene (plasmid 1791; [35]). Lysates of cells transfected with the empty vector pECE (mock-control) (1) or pECE Flag-SIRT1 vector (2) were used for Western Blot analysis. B) mock-transfected (mock-control) and Flag-SIRT1 transfected HepG2 cells were stimulated with resveratrol [50, 100 µM Resv.] for 24 h and Western Blot analysis of acetylated p53 (K382), p21 and GAPDH was performed. Densitometric anaylsis of acetylated p53 of three independent Western Blots is shown. Data were normalised to non-transfected HepG2 cells stimulated with resveratrol alone which was set 1. C) To analyse the effect of SIRT1 overexpression on resveratrol-induced NAMPT release, supernatant of mock-transfected and Flag-SIRT1 transfected HepG2 cells stimulated with or without resveratrol [100 µM] were used to measure eNAMPT level. One representative Western blot out of 3 independent experiments is shown. D) Cell viability of mock-transfected and Flag-SIRT1 transfected HepG2 cells treated with resveratrol [100 µM] (black bars) was measured using WST-1 assay (n = 3). Data were normalised to untreated mock-control which was set 1. E) mock-transfected (white bars) and Flag-SIRT1 transfected HepG2 cells (black bars) were stimulated with resveratrol [25, 50 µM] for 24 h. Percentage of cells in the S-phase were measured by PI staining and FACS analysis. All data are shown as mean± SEM (n = 4). The difference between two groups was evaluated using unpaired Student’s *t*-test (##p<0.01 mock-transfected cells compared to mock-transfected cells treated with resveratrol (white bars, mock-control), **p<0.01, ***p<0.001 Flag-SIRT1 transfected cells treated with resveratrol (black bars) compared to resveratrol-treated mock-transfected cells (white bars).

Since resveratrol and NMN co-treatment did not augment resveratrol-induced p53 hyperacetylation in hepatocarcinoma cells, we tried to overcome this effect by transiently overexpressing SIRT1 in HepG2 cells (Fig. 9A). Our data revealed that SIRT1 overexpression significantly decreased resveratrol- induced p53 hyperacetylation ([50 µM] −76.6±6.5%, [100 µM] −69.9±15.9%, p<0.05) and its transcriptional activity in HepG2 cells (Fig. 9B). We then investigated whether a SIRT1 overexpression would be able to abrogate resveratrol-induced NAMPT secretion in HepG2 cells. SIRT1 overexpressing HepG2 cells treated with 100 µM resveratrol led to decreased eNAMPT levels in the supernatant compared to mock-transfected cells treated with resveratrol alone (Fig. 9C). This suggests that SIRT1 may play a crucial role in the mechanism of resveratrol-induced NAMPT secretion. Reduction in cell viability upon resveratrol treatment [100 µM] was not abolished by SIRT1 overexpression (Fig. 9D) indicating that apoptosis inducing factors were still activated and not dependent on SIRT1. However, the resveratrol- induced cell cycle arrest in the S-phase was significantly decreased after SIRT1 overexpression ([con] 7.4±0.9%, [con+25 µM] 28.1±2.6%, [Flag-SIRT1+25 µM] 18.5±3.8%; [con+50 µM] 26.6±3%, [Flag-SIRT1+50 µM] 17.1±3.2%) (Fig. 9E).

## Discussion

There is growing *in vitro* and *in vivo* evidence demonstrating the inhibitory effects of resveratrol on liver cancer [44]–[46]. It is known that resveratrol affects numerous signal transduction pathways associated with tumorigenesis [47]. However, the mechanisms how resveratrol selectively modulates proliferation and apoptosis in tumor cells are not fully understood. A recent study demonstrated that resveratrol has the chemical structure to inhibit the activity of different human histone deacetylases (HDACs), important transcriptional and post-translational regulators [48]. We investigated the molecular mechanisms of resveratrol-induced reduction of cell viability in human hepatocellular carcinoma cells and compared the results with non-cancerous primary human hepatocytes. We found that resveratrol selectively induced apoptosis in HepG2 and Hep3B cells, but not in primary hepatocytes. Our data confirm the apoptotic effects of resveratrol on hepatocarcinoma cells independent of p53 function. Furthermore, several other studies reported that resveratrol induced p53-independent apoptosis in tumor cells [49], [50], indicating that p53 is not an absolute requirement for the apoptotic effect of resveratrol. In contrast, we found an arrest of cells in the S- and G2/M-phase of the cell cycle only in p53 wild-type HepG2 cells and not in Hep3B cells lacking p53, which was also shown by other groups [7], [51], [52].

Our study revealed that NAMPT and SIRT1 were expressed in an opposite way in hepatocarcinoma cells and primary hepatocytes and were differentially regulated by resveratrol. Other groups found SIRT1 expression to be significantly elevated in hepatocellular carcinoma (HCC) compared to non-cancerous tissues, the expression levels correlated with tumor grades and predicted poor prognosis. SIRT1 was shown to promote tumorigenesis in HCC, and inhibition of SIRT1 consistently suppressed the proliferation of HCC cells *in vitro* or *in vivo* via the induction of cellular senescence or apoptosis [53]–[56]. The expression and role of NAMPT in HCC has not been characterised so far. In contrast to findings in other cancer cell types [18], we found that hepatocarcinoma cells express lower levels of NAMPT compared to non-cancerous primary hepatocytes. However, we found that HepG2 cells possess a higher basal NAMPT activity than primary hepatocytes, which goes in line with the comparable NAD levels in both cell types despite weaker NAMPT expression in hepatocarcinoma cells.

Under basal conditions primary hepatocytes released higher amounts of NAMPT into the supernatant than HepG2 cells. As shown by our group, HepG2 cells and primary human hepatocytes constitutively release NAMPT in its dimeric, enzymatically active form [43]. Presumably, due to the higher amount of cellular NAMPT protein in primary human hepatocytes compared to hepatocarcinoma cells, NAMPT is constitutively more released from primary hepatocytes leading to higher basal eNAMPT level. However, we cannot completely preclude, that due to necrosis of fragile or dead primary hepatocytes, proteins are released into the supernatant.

There are several reports showing that resveratrol acts as a natural SIRT1 activator [10], [57]–[60]. We observed increased NAMPT activity and intracellular NAD levels in primary hepatocytes providing evidence for resveratrol as SIRT1 activator in non-cancerous cells. However, recent data showed that resveratrol is not a direct activator of SIRT1 and therefore some mediators are may be involved in this interplay [48], [61]–[63]. Moreover, little is known about NAMPT and SIRT1 regulation by resveratrol in cancer cells. A recent report showed that SIRT1 inhibition is involved in resveratrol-induced cell death in Hodgkin lymphoma (HL)-derived L-428 cells [11]. Additionally, neuroblastoma cells treated with resveratrol also underwent apoptosis and showed a downregulaton of SIRT1 [64]. In our study, resveratrol-stimulated HepG2 cells showed similar responses like cells treated with the SIRT1 inhibitor EX527. Thus, we assume that resveratrol is rather acting as a NAMPT and SIRT1 inhibitor in hepatocarcinoma cells. However, there are several reports demonstrating other mechanisms leading to p53 acetylation by resveratrol [65], [66].

Our findings indicate that NMN did not ameliorate resveratrol-induced effects on apoptosis, cell cycle arrest and NAMPT release, suggesting that the availability of NAD is not a limiting factor in this scenario. A variety of posttranslational modifications in SIRT1 N- and C-terminal extensions have been reported, effecting SIRT1 enzyme activity and protein interactions [67], [68]. Our findings raise the possibility that, in some cases, the regulation of SIRT1 by other proteins may be more important than NAD availability. Increase in p53 transcriptional activity and induction of S-phase arrest observed upon treatment with resveratrol were abrogated upon SIRT1 overexpression. However, SIRT1 overexpression was not able to augment reduced cell viability in HepG2 cells under high resveratrol concentrations. Resveratrol affects a multitude of other signal transduction pathways associated with apoptotic mechanisms and transcriptional regulation [66], [69] that are still activated and not SIRT1 dependent [64]. Thus, these collective activities, rather than just a single effect, may account for the anticancer properties of resveratrol. However, our data give evidence that resveratrol regulates NAMPT activity in cancer cells and non-cancerous cells. Resveratrol could regulate NAMPT enzymatic activity by at least two hypothetical mechanisms: i) by direct interaction inducing conformational changes that lead to alterations of enzymatic activity, or ii) by inducing a posttranslational modification of NAMPT. Our study revealed that SIRT1 inhibition downregulates NAMPT activity and induces NAMPT secretion. This provides the basis for further mechanistic studies on NAMPT-SIRT1 interaction and their regulation.

Further, we found a time- and dose-dependent NAMPT release after resveratrol stimulation of HepG2 cells which was associated with increased *NAMPT* mRNA expression. The association of increased NAMPT release and mRNA expression has also been shown by Kover *et al*. in human islets [70]. eNAMPT has been described to act as a cytokine (as pre-B cell colony enhancing factor, PBEF) [71] or as an adipokine (visfatin) [72], [73] but also has extracellular enzymatic function to yield NMN [74]. To our knowledge, for the first time our data point to SIRT1 as regulator of NAMPT secretion and NAMPT enzymatic activity in the supernatant. We could show that the resveratrol-induced NAMPT release was significantly reduced after SIRT1 overexpression, indicating a crucial role for SIRT1 in resveratrol-mediated NAMPT secretion.

In summary, our study revealed that resveratrol selectively induced p53-independent cell death in hepatocarcinoma cells and differentially regulated NAMPT and SIRT1 in cancer cells and non-cancerous cells. Our data give evidence that in contrast to normal hepatocytes, resveratrol does not act as a NAMPT and SIRT1 activator in hepatocarcinoma cells. However, it remains to be investigated whether NAMPT interacts with SIRT1 and how it is regulated by resveratrol or other mediators and linked to cellular metabolism and apoptosis. This will provide novel insights concerning the potential of NAMPT and SIRT1 as therapeutic targets in hepatocellular carcinoma.

## Supporting Information

Figure S1 **Establishment of parameters for a NAMPT enzymatic assay.** Assay conditions, such as A) protein amount, B) pH value and C) incubation time were optimized for measuring NAMPT enzymatic activity. D) We validated the assay performance by adding the specific NAMPT inhibitor FK866 to the lysate before measuring NAMPT activity. As expected, FK866 induced a dose-dependent decrease in NAMPT activity with an IC_50_ value of 8.2 nM. Experiments were performed in HepG2 cells. Data are presented as mean± SEM.
(TIF)

Figure S2 **Cell cycle arrest in Hep3B and a representative dot plot of HepG2 cells.** A) PI staining of cell cycle distribution of Hep3B cells (n = 2) stimulated with different concentration of resveratrol [25/50/100 µM] for 24 h. B) A representative dot plot of cell cycle analysis of HepG2 cells. The left plot shows pulse width versus area; this is the plot used to distinguish between single cells and aggregates. Single cells have been gated and a FL2-Area histogram has been drawn and formatted to show only the events inside of the single cell region.
(TIF)

Figure S3 **Resveratrol does not have cytotoxic effects on HepG2 cells and primary human hepatocytes.** HepG2 cells and primary human hepatocytes were stimulated with resveratrol [10/25/50/100 µM] in serum-free medium for 24 h and supernatant was used for the ToxiLight Non-destructive Cytotoxicity BioAssay. A) Primary human hepatocytes (n = 3) and B) HepG2 cells (n = 3) showed no cytotoxic effects after stimulation with resveratrol. Data are shown as mean± SEM. Statistical analysis was performed using one-way ANOVA and the Bonferroni post hoc test (n.s. not significant). C) Supernatants of resveratrol [100 µM] or serum-free medium (con) treated HepG2 cells after 6, 12 and 24 h were used to measure extracellular NAMPT levels by Western Blot.
(TIF)

Figure S4 **Resveratrol downregulates NAMPT enzymatic activity in Hep3B cells.** Hep3B cells were stimulated with resveratrol [10/25/50/100 µM] in serum-free medium for 24 h. NAMPT enzymatic activity was measured by the conversion of ^14^C- labelled nicotinamide to ^14^C-NMN (see Material and Methods). Counts (cpm) were normalised to µg total protein in each sample measured by BCA protein assay. Data are represented as mean± SEM and statistical analysis was performed using one-way ANOVA and the Bonferroni post hoc test (*p<0.05).
(TIF)

Figure S5 **p21 and Bax expression in HepG2 cells and primary human hepatocytes.** HepG2 cells and primary human hepatocytes were stimulated with resveratrol [10/25/50/100 µM] in serum-free medium (0) for 24 h. *p21* mRNA expression in A) HepG2 cells (n = 3) and B) primary human hepatocytes (n = 4). C) Lysates of HepG2 cells (n = 3) were used for Western Blot analysis of Bax protein expression. GAPDH was used as loading control. One representative blot out of 3 independent experiments is shown.
(TIF)

Figure S6 **NMN does not ameliorate resveratrol-mediated effects on cell viability, NAMPT activity, NAMPT release and p53 hyperacetylation.** Given that resvertarol down-regulates NAMPT and increases p53 acetylation in hepatocarcinoma cells which was absent in primary hepatocytes we hypothesised that the administration of NMN, the reaction product of NAMPT and a precursor of NAD, is able to ameliorate the resveratrol-mediated effects by increasing SIRT1 activity. At the beginning, we tested whether HepG2 cells are able to utilize exogenous NMN [500 µM] and to synthesize NAD. Therefore, we stimulated the cells with FK866 [10 nM] to inhibit NAMPT activity and co-stimulated the cells with NMN [500 µM]. We could show that FK866 depleted the intracellular NAD levels by −79.4±3.3% in HepG2 cells which could be restored by NMN supplementation. A) NAD levels of HepG2 cells (n = 5) treated with the NAMPT inhibitor FK866 [10 nM] (white bars) in serum-free medium (con) and NMN [500 µM] (black bars) for 24 h. Annexin V/PI apoptosis assay of B) HepG2 cells (n = 3) and C) Hep3B cells (n = 2) treated with resveratrol [25/50/100 µM] in serum-free medium and co-stimulated with NMN [500 µM] for 24 h. An+ and An+/PI+ cells were considered apoptotic. Data are represented as mean± SEM. Differences between two groups were evaluated using unpaired Student’s *t*-test (resvertarol (white bar) compared to resveratrol +NMN (black bar)). Then D) Western Blot analysis of cleaved caspase-3, E) acetylated p53 (K382) and F) eNAMPT levels in supernatant of these cells were performed. One representative blot out of at least 3 independent experiments is shown.
(TIF)
