# Supplementary figures and images for: Resveratrol Differentially Regulates NAMPT and SIRT1 in Hepatocarcinoma Cells and Primary Human Hepatocytes
Source: PLoS One. 2014 Mar 6;9(3):e91045. doi: 10.1371/journal.pone.0091045 (PMC3946349; doi:10.1371/journal.pone.0091045)

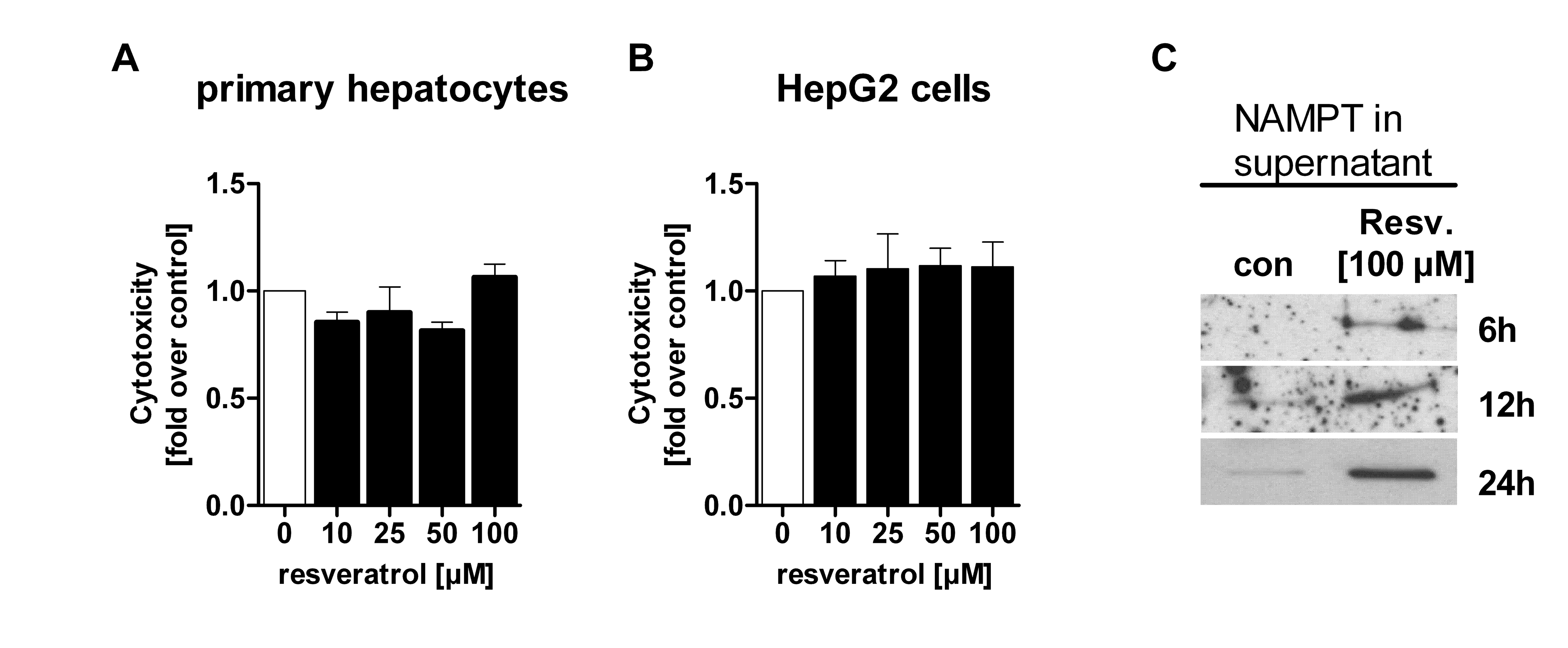

Supplement: Figure S3 — Resveratrol does not have cytotoxic effects on HepG2 cells and primary human hepatocytes. HepG2 cells and primary human hepatocytes were stimulated with resveratrol [10/25/50/100 µM] in serum-free medium for 24 h and supernatant was used for the ToxiLight Non-destructive Cytotoxicity BioAssay. A) Primary human hepatocytes (n = 3) and B) HepG2 cells (n = 3) showed no cytotoxic effects after stimulation with resveratrol. Data are shown as mean± SEM. Statistical analysis was performed using one-way ANOVA and the Bonferroni post hoc test (n.s. not significant). C) Supernatants of resveratrol [100 µM] or serum-free medium (con) treated HepG2 cells after 6, 12 and 24 h were used to measure extracellular NAMPT levels by Western Blot. (TIF) [file pone.0091045.s003.tif]

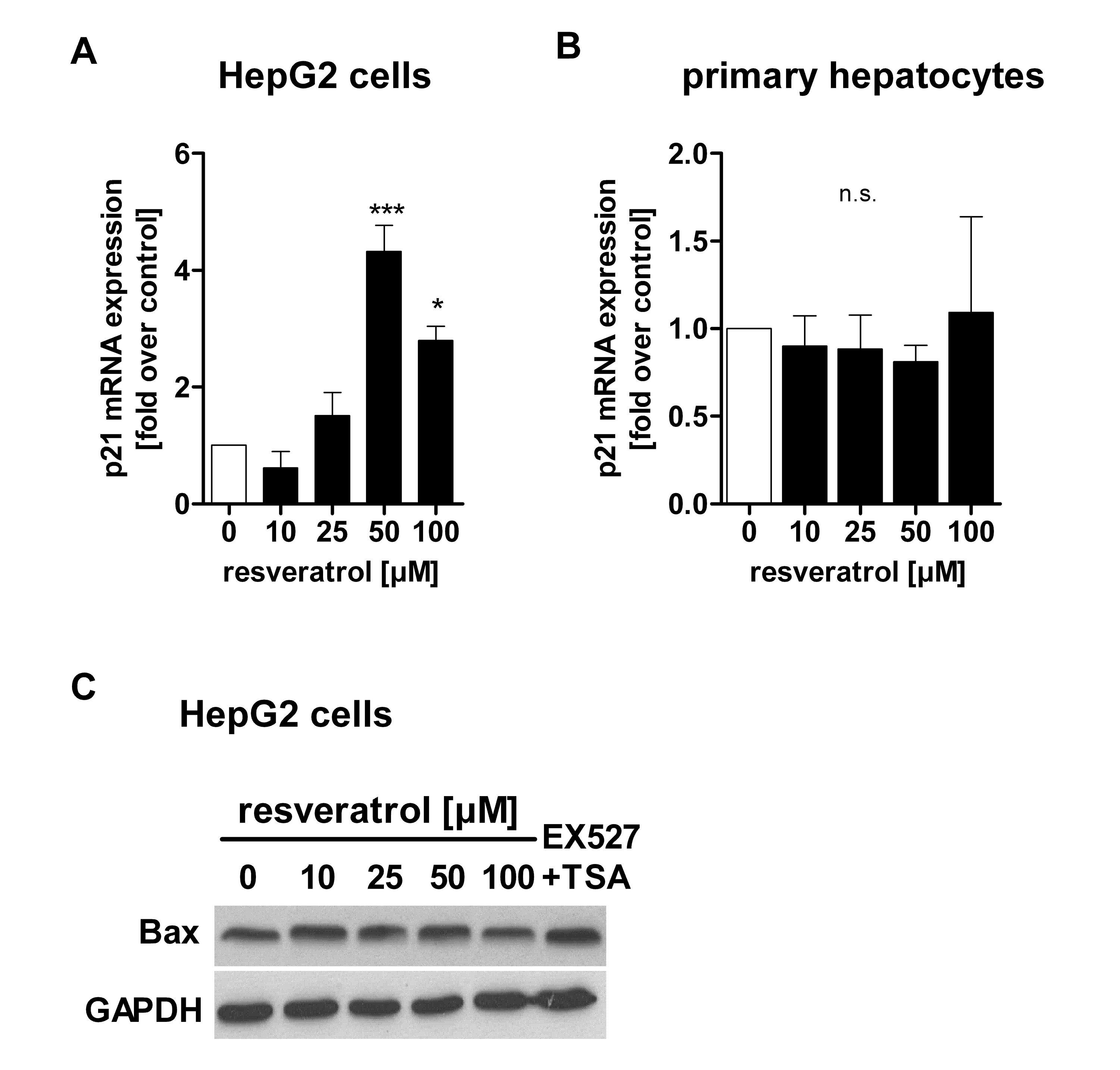

Supplement: Figure S5 — p21 and Bax expression in HepG2 cells and primary human hepatocytes. HepG2 cells and primary human hepatocytes were stimulated with resveratrol [10/25/50/100 µM] in serum-free medium (0) for 24 h. p21 mRNA expression in A) HepG2 cells (n = 3) and B) primary human hepatocytes (n = 4). C) Lysates of HepG2 cells (n = 3) were used for Western Blot analysis of Bax protein expression. GAPDH was used as loading control. One representative blot out of 3 independent experiments is shown. (TIF) [file pone.0091045.s005.tif]
